# Tracheotomy and Intubation

**Published:** 1905-01-21

**Authors:** 


### TRACHEOTOMY AND INTUBATION.

The respective advantages and disadvantages of tracheotomy and intubation were well explained in a recent clinical lecture at Guy's Hospital by Mr. Stewart. He held that intubation was the operation of choice, and that the objections to it were to some extent imaginary and to some extent based upon cases of laryngeal obstruction due to diphtheria in the days before antitoxin. One of the merits of intubation is the ease with which it may be performed. Thus it takes only about five seconds to perform, while, however much experience a man has had, tracheotomy on a small child is always exceedingly difficult, and may take a long time to complete. Again, the parents will consent more readily to intubation than to tracheotomy, and the practitioner will be more disposed to carry out the necessary interference at an earlier period in the case of intubation, and so the child will be relieved before its condition has advanced too far. On account of the objection to operation, tracheotomy is often put off longer than it should be, so that the child does not recover. Mr. Stewart also affirms that broncho-pneumonia is a sequel less common after intubation than tracheotomy, in diphtheria at any rate, and that there is a lower mortality after the former operation than after the latter. The chief objections urged against intubation are (1) that membrane may become dislodged and block the tube; (2) that the tube may be coughed out; and (3) that there is a danger of causing laryngeal stenosis. Mr. Stewart deals seriatim with these objections. The tube should be removed immediately if it becomes blocked with membrane, and the nurse in attendance should be instructed to pull the tube out if the child becomes blue and cannot breathe. If the tube is coughed out there is no immediate danger as a rule. The instant suffocation, which is supposed to follow, is to a large extent imaginary. Out of 40 cases Mr. Stewart has met with no troubles at all in connection with the coughing out of the tube or blocking with membrane. The danger of larygeal stenosis may be avoided by not leaving in the tube too long. Mr. Stewart advises that if for some reason the child has not recovered sufficiently to do without the tube at the end of the fifth or sixth day, tracheotomy should be performed; the danger of stenosis will then be circumvented.

